# A questionnaire using vocal symptoms in quality control of phonosurgery: vocal surgical questionnaire

**DOI:** 10.1186/s12901-018-0057-0

**Published:** 2018-06-15

**Authors:** Aleksander Grande Hansen, Chi Zhang, Jens Øyvind Loven, Hanne Berdal-Sørensen, Magnus TarAngen, Rolf Haye

**Affiliations:** 1 Department of Ear, Nose and Throat, Head and Neck Surgery, Lovisenberg Diaconal Hospital, Oslo, Norway; 2 Institute of Basic Medical Sciences, Faculty of Medicine, University of Oslo, Oslo, Norway; 3 Institute of Clinical Medicine, Faculty of Medicine, University of Oslo, Oslo, Norway

**Keywords:** Laryngology, Questionnaire, Voice assessment, Phonosurgery, Visual analogue scale, Likert scale

## Abstract

**Background:**

Quality control after phonosurgery is important and may be time consuming. Often questionnaires focusing on quality of life are applied. We aimed at investigating the use of organ specific symptoms, such as hoarseness and voice failure with the use of self-reported visual analogue scales (VAS) and Likert-scales.

**Methods:**

A vocal surgical questionnaire using VAS and Likert-scales for hoarseness, voice failure and factors that could influence voice quality was given twice consecutively to a group of healthy volunteers (*n* = 57, 45 female) and a group of voice patients (*n* = 34, 21 females) for a test/re-test study. Secondly, a group of patients undergoing surgery (*n* = 90, 61females) answered the questionnaire preoperatively and postoperatively. The difference between test/retest, healthy volunteers and patients, and between pre- and postoperative results were compared.

**Results:**

There was no significant difference in the test/retest results in healthy volunteers nor in the patient group. There was statistically significant difference between the healthy volunteers and patients, and between the preoperative and postoperative results after phonosurgery.

**Conclusion:**

This short and organ specific questionnaire clearly demonstrates the effect of phonosurgery, making it an easy and relevant tool in quality control and potentially reducing the need of postoperative controls in the outpatient clinic.

## Background

Quality control after treatment of vocal disorders is often implemented using mailed questionnaires [1–5]. Most of them focus on quality of life items [6]. Surgeons treating laryngeal lesions are more interested in organ specific vocal symptoms, particularly hoarseness and voice failure, as these symptoms often provide indication for surgery and are considered important in assessing the results of phonosurgery. Hoarseness is a symptom describing a vocal change, e.g. a breathy, creaky or raspy voice. Voice failure describes that the voice “gives out” in the middle of speaking. Surgeons also want to be informed of any change in symptom load, other treatments, occupational as well as social habits that may influence treatment. Ideally, all patients undergoing phonosurgery should be recalled for a postoperative consultation with stroboscopy, but this is challenging in terms of human and financial resources. A clinical postoperative questionnaire would allow to only recall patients with persistent symptoms. Our aim, therefore, was to construct a questionnaire focusing on hoarseness and voice failure, using visual analogue scales (VAS) to compare these symptoms between healthy volunteers and patients, and between preoperative and postoperative symptom load.

## Methods

This study was performed at the Department of Oto-Rhino-Laryngology, Head and Neck Surgery of Lovisenberg Diaconal Hospital in Oslo, Norway. The study was approved by the Ethics Committee at the hospital.

### Vocal surgical questionnaire (VSQ)

**Fig. 1:**
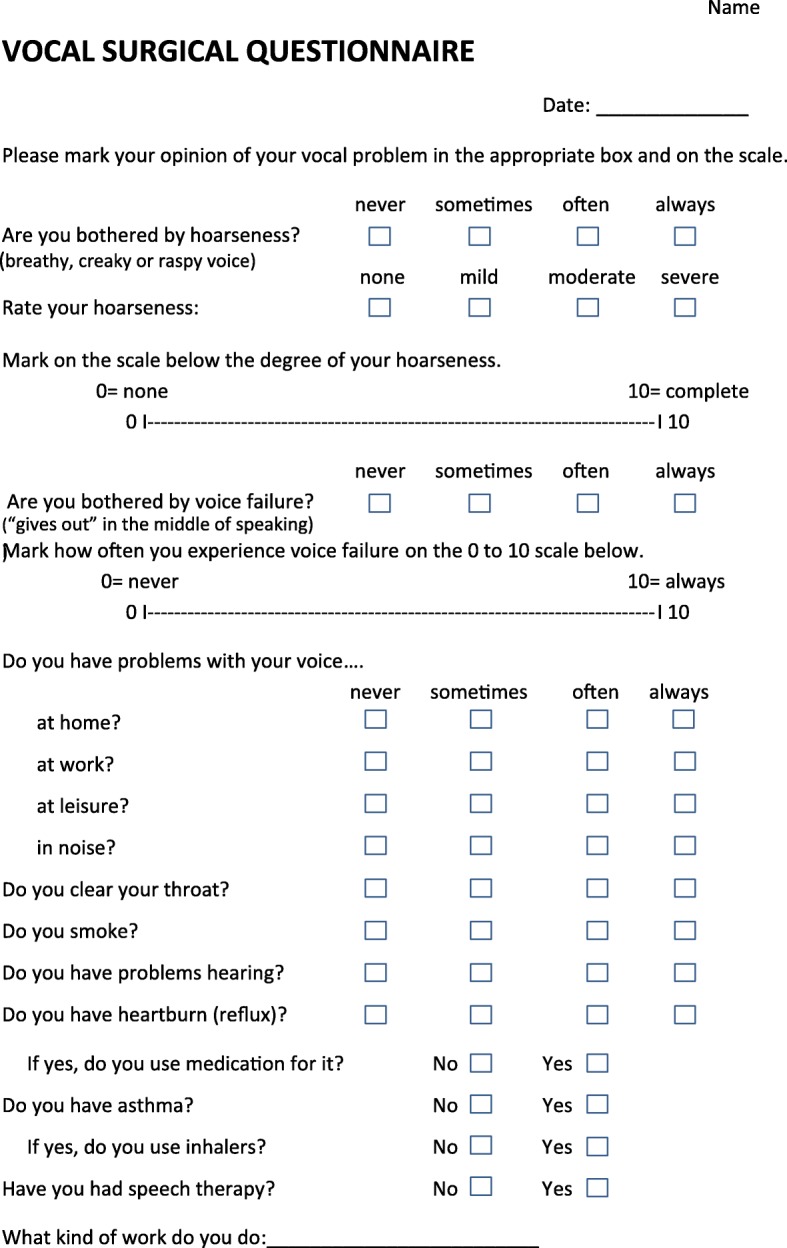
The vocal surgical questionnaire (VSQ) used in the preoperative recording of vocal symptoms

We constructed a VSQ for a preoperative resume of the patient’s symptoms and the relevant clinical data. The preoperative version of the VSQ was twice presented to patients and controls as a test-retest study. In the second presentation we asked if there had been a change in the vocal function since the first response. If there had been a change this test-retest sample was discarded. The preoperative version of the VSQ (Fig. 1) consists of one VAS for hoarseness and another one for voice failure. Both VAS were 10 cm long, marked 0 (= no hoarseness/voice failure) on the left end, and 10 (= complete hoarseness/voice failure) on the right end. The patients were asked to rate their subjective sense of hoarseness and voice failure by putting a mark on the scale. The score was measured in millimetres (mm) from the left end of the scale to this mark.

We also included four point Likert scales for hoarseness and voice failure. The grades were 0 = none/never, 1 = mild/sometimes, 2 = moderate/often and 3 = severe/always. Four point Likert scales were also used in assessing vocal function in different social settings: at home, at work, during leisure, in noisy environment with the options 0 = never, 1 = sometimes, 2 = often, 3 = always. The patients were asked about how often they needed to clear their throat, their smoking habits, hearing disability, reflux symptoms, asthma and use of related medication. The final items were related to occupation and the use of speech therapy.

The postoperative version of the VSQ contained the same questions as the preoperative one with an additional item about the overall improvement in the voice after surgery. The postoperative version of the VSQ was mailed to the patients 4 months postoperatively together with a cover letter and a pre-paid return envelope.

### Subjects

The study population consisted of three groups: controls, i.e. persons without a voice problem, patients included for the test-retest study and an expanded group of patients treated surgically. Persons/patients with an inadequate command of the Norwegian language were excluded.

Healthy volunteers were recruited from different departments at our hospital. They could not complain of voice disorders. The volunteers twice responded to the preoperative version of the VSQ with a minimum time interval of 1 week. To ensure that the two responses evaluated the same vocal function, there should not be any change in vocal function in the time interval between the two responses. They were given a study identification number only known to one of the investigators. The lists with the identification numbers were subsequently destroyed after the responses were obtained.

Patients referred to the department for benign laryngeal diseases were asked to participate in a test-retest study of the preoperative version of the VSQ. The time interval was a minimum of 1 week. Patients with malignant disorders were excluded. To ensure that the two responses evaluated the same vocal function, participants with changes in the vocal function between the two responses were excluded.

Patients with benign laryngeal disorders were asked to respond to the VSQ and also to the postoperative version of the VSQ after 4 months. We included patients with laryngeal papillomatosis, vocal sulcus, atrophic vocal cords, recurrent nerve palsy and spastic dysphonia. Surgery was performed during general anaesthesia. Benign laryngeal lesions were treated microscopically with microsurgical instruments or laser, spastic dysphonia with injections of botulinum toxin and vocal sulcus lesions and atrophic vocal cords with injections of hydroxyapatite.

#### Statistical analyses

On test-retest studies, the mean and variance of VAS were calculated for both questionnaires. The difference between the answers from the two questionnaires of the same cohort was compared with Wilcoxon signed rank test. Cohen’s kappa was computed on test-retest cohorts to verify the reliability of the questionnaire. Cronbach’s alpha was computed on the same cohorts to quantify the internal consistency among questions. We used Spearman’s correlation coefficient to quantify the correlation between VAS and Likert scale of hoarseness and voice failure both on pre- and postoperative cohorts. Wilcoxon signed rank test was used to compare the difference between responses to Likert scale questions pre- and postoperatively. All statistics were performed using R, version 3.4.2, with package “psych”.

## Results

### Controls, test-retest

We recruited 57 healthy volunteers (45 females and 12 males) with a mean age of 48.6 years. There was no significant difference in VAS scores of hoarseness and voice failure between their first and second response to the VSQ (Table 1). The ratings between the two responses to vocal function in different social environments, hearing loss, asthma, regurgitation and clearing of the throat were not significantly different (Table 2).

**Table 1:** VAS scores (Standard Deviation) for control group and patient test-retest; comparison between patients and controls and comparison between pre- and postoperative results

Control group				
	1. response	2. response	Difference	*p*-value
Hoarseness	4.83 (12.25)	4.73 (10.57)	0.74	0.63
Voice failure	1.91 (3.84)	2.22 (4.19)	−0.98	0.70
Patient test-retest				
	1. response	2. response	Difference	p-value
Hoarseness	70.64 (24.49)	71.74 (19.26)	−0.39	0.89
Voice failure	49.09 (30.06)	54.78 (26.72)	−3.25	0.92
Comparison patients vs. controls using mean of 1. and 2. response				
	Patients	Controls	Difference	*p*-value
Hoarseness	71.19 (22.03)	4.78 (11.44)	66.41	< 0.0001
Voice failure	51.85 (28.44)	2.07 (4.02)	49.78	< 0.0001
Pre and postoperative results compared				
	Preoperative	Postoperative	Difference	*p*-value
Hoarseness	64.25 (23.20)	23.89 (26.78)	41.17	< 0.0001
Voice failure	43.82 (27.53)	17.95 (25.34)	26.08	< 0.0001

**Table 2:** Comparison between responses of first and second questionnaire in controls (volunteers) and patients

	Controls	Patients
Vocal function		
At home	0.13	0.34
At work	0.10	0.50
In noise	0.47	0.26
At leisure	0.21	0.36
Hawking	0.40	0.40
Smoking	0.55	0.48
Hearing problem	0.58	0.75
Reflux	0.36	0.52
Reflux medication	0.57	0.87
Asthma	0.75	0.65
Asthma spray	0.61	1.00

Cohen’s kappa

### Patients, test-retest

Thirty-four patients (21 females and 13 males) with a mean age of 43.5 years twice responded to the preoperative version of the VSQ. There were six smokers and two patients with asthma. No significant difference was found between the first and second responses to the VAS scores of hoarseness and voice failure (Table 1). Cohen’s kappa was computed for Likert scores of vocal function in different social settings, social habits, illnesses and treatments to verify the reliability of the questionnaire and the results were positive (Table 2). The Cronbach’s alpha tests for questions of voice function in different social settings showed high values for both the first and second questionnaire (Table 3).

**Table 3:** Reliability of 1. and 2. questionnaire in patients regarding voice failure in different social settings

	1. questionnaire	2. questionnaire
All items	0.89	0.91
At home	0.85	0.85
At work	0.85	0.91
In noise	0.90	0.89
At leisure	0.83	0.85

Chronbach alpha

### Comparison between controls and patients

VAS scores of hoarseness and voice failure (using the average of the first and second questionnaire) showed significant differences between patients and controls (Table 1).

### Results of surgery

We compared the pre and postoperative data of 90 patients (29 males and 61 females) with a mean age of 47.2 years who were surgically treated of benign vocal cord disorders. All patients from the test-retest study were included in the study of the surgical results. We recorded 15 smokers and 11 patients with asthma. The VAS scores for hoarseness and voice failure were significantly different between the pre- and postoperative recordings (Table 1).

**Fig. 2:**
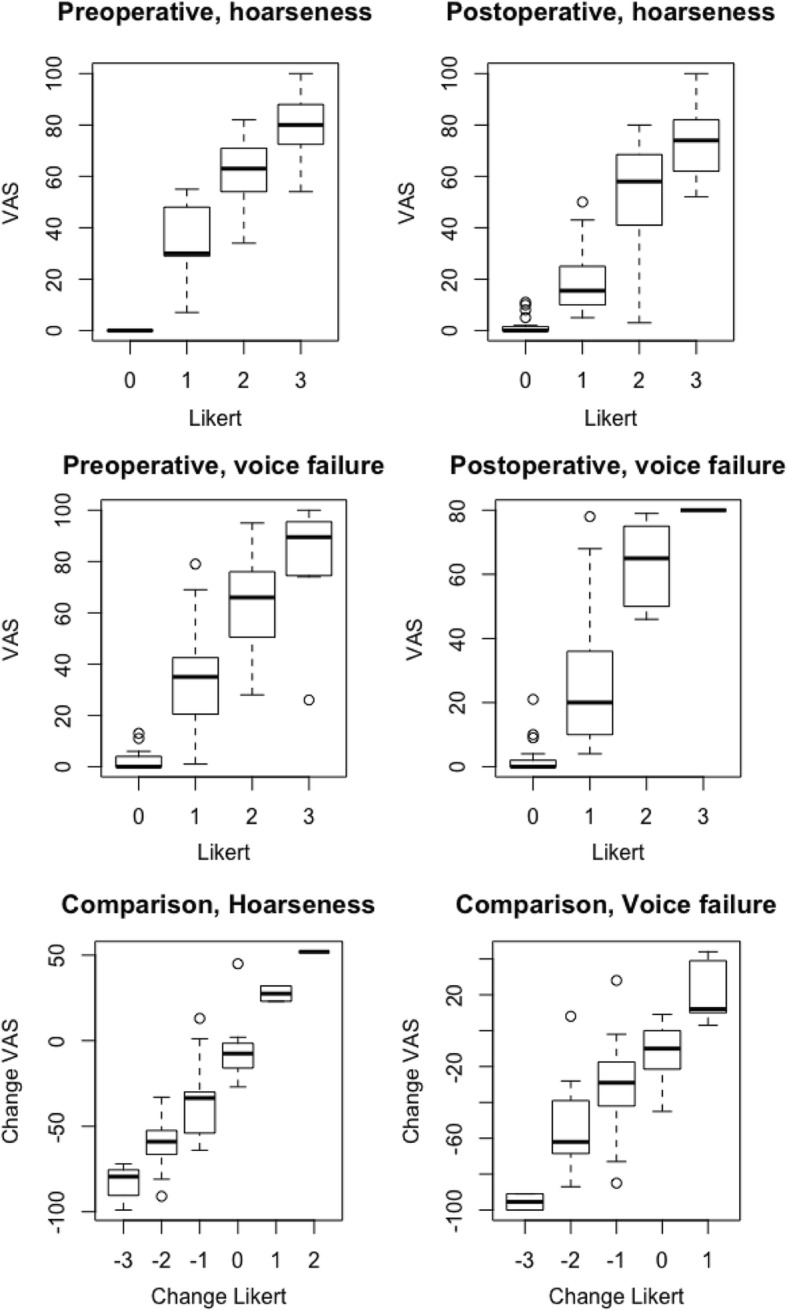
Comparison between visual analogue scale (VAS) and Likert scores for hoarseness and voice failure, pre-, postoperative and improvement

We found that the Likert and VAS scores for hoarseness and voice failure were highly correlated both for the pre- and postoperative recordings and the differences between them using Spearman’s correlation (Table 4). This is illustrated in Fig. 2.

**Table 4:** Correlation between VAS and Likert scale for pre- and postoperative patients.

	Preoperative	Postoperative	Comparison between pre and post
Hoarseness	0.76	0.91	0.85
Voice failure	0.81	0.87	0.69

Spearman’s correlation

The Likert scores (using median values) before and after surgery and their differences for vocal function in social settings, hearing problems, smoking, regurgitation, clearing of throat, asthma and treatments are shown in Table 5. Patients reported significant improvement in all items except for smoking, hearing problems, heartburn and asthma.

**Table 5:** Comparison of Likert scores for vocal symptoms, vocal function, social habits, illness and treatments between pre-, postoperative and change in ratings

	Median pre	Median post	Median change	*P*-value
Hoarseness	2	1	−1	< 0.0001
Voice failure	1	0	− 1	< 0.0001
Hoarseness at home	2	0	−1	< 0.0001
At work	2	0	−1	< 0.0001
At leisure	2	0	−1	< 0.0001
In noise	2	1	−1	< 0.0001
From others	2	0	−1	< 0.0001
Clear throat	1	1	−1	< 0.0001
Smoking	0	0	0	0.032
Problem hearing	0	0	0	0.937
Heartburn	0	0	0	0.44
Asthma	0	0	0	0.9772

Wilcoxon signed rank test

## Discussion

We have assessed the VSQ for use as an instrument in quality control of phonosurgery. The test-retest of controls and of patients did not show any significant change in hoarseness or voice failure when the questionnaire was twice applied to the participants. There was a statistically significant difference in the results between patients and controls for hoarseness, voice failure and vocal function in different social settings. The postoperative results showed a significant improvement in hoarseness, voice failure and vocal function. We therefore believe that our findings could make the VSQ a useful instrument in quality control of phonosurgery.

Studies have shown that short questionnaires give better response rates than longer ones [7]. We, therefore, intended to remove overlapping questions. The scores for hoarseness and voice failure which were recorded both on Likert scales and VAS were comparable. As VAS is a continuous and Likert an interrupted scale we prefer to only use VAS for these items. The VSQ has four different questions about the voice quality in different social settings. As there was no significant difference in improvement after surgery between the different settings, we believe that one item should be sufficient to describe the social aspect of voice function. The voice quality at home was the only one responded to by all patients and therefore best suited for our purpose. Professional voice users could benefit from the evaluation of vocal symptoms in different social settings. Therefore, these questions could remain in the VSQ for professional voice users.

The postoperative responses to hearing problems, asthma, smoking habits, regurgitation and use of medication were only marginally different from the preoperative ones. We, therefore, expect that most of the postoperative responses of these items will remain unchanged. Thus, one open-ended question of any change in smoking habit, hearing, heart burn, asthma, treatments and occupation would be sufficient. The question about speech therapy after surgery should remain. The postoperative questionnaire could thereby be reduced to eight items.

There are several questionnaires in use for assessing the status of the voice before and after treatment [8], and objective measurements often do not correlate with self-assessed voice symptoms [9]. Questionnaires often pose questions on voice impairment (vocal physical symptoms), voice function and the impact of the voice on the patients’ emotional wellbeing. Most questionnaires use a five point Likert scale for each of the questions [10] or VAS [11, 12]. The scores are added for a final result. Each question has equal merit. We wanted to focus on the two main physical aspects of the voice and in addition on the medical conditions and therapies, social habits and occupation that may influence the voice. These are important in relation to surgery. Changes in these items may be contributory to improvement or deterioration of the voice, thus they have a natural place in a vocal questionnaire.

The vocal function is important for the patient’s emotional well-being, social function and occupation. However, questionnaires do not evaluate the impact of emotions on the vocal function and we acknowledge that changes in the emotions in the time period between the pre- and postoperative questionnaires could have influenced our results.

## Conclusions

We believe that this short postoperative questionnaire focusing on hoarseness and voice failure gives a satisfactory assessment of the patient’s response to phonosurgery. This will help us decide whether to recall the patient for a new consultation or not. A satisfactory response will obviate the need of a recall and save time for other patients.

## Abbreviations

VAS: Visual analogue scale
VSQ: Vocal surgical questionnaire

## References

[1] Laukkanen AM, Leppanen K, Ilomaki I (2009). Self-evaluation of voice as a treatment outcome measure. Folia Phoniatr Logop.

[2] Uloza V (1999). Effects on voice by endolaryngeal microsurgery. Eur Arch Otorhinolaryngol.

[3] Aaby C, Heimdal JH (2013). The voice-related quality of life (V-RQOL) measure--a study on validity and reliability of the Norwegian version. J Voice.

[4] Pernambuco L, Silva MP, Almeida MN, Costa EB, Souza LB (2017). Self-perception of swallowing by patients with benign nonsurgical thyroid disease. Codas.

[5] Karlsen T, Grieg AR, Heimdal JH, Aarstad HJ (2012). Cross-cultural adaption and translation of the voice handicap index into Norwegian. Folia Phoniatr Logop.

[6] Branski RC, Cukier-Blaj S, Pusic A, Cano SJ, Klassen A, Mener D, Patel S, Kraus DH (2010). Measuring quality of life in dysphonic patients: a systematic review of content development in patient-reported outcomes measures. J Voice.

[7] Rosen CA, Lee AS, Osborne J, Zullo T, Murry T (2004). Development and validation of the voice handicap index-10. Laryngoscope.

[8] Birkent H, Sardesai M, Hu A, Merati AL (2013). Prospective study of voice outcomes and patient tolerance of in-office percutaneous injection laryngoplasty. Laryngoscope.

[9] Ma EP, Yiu EM (2001). Voice activity and participation profile: assessing the impact of voice disorders on daily activities. J Speech Lang Hear Res.

[10] Deary IJ, Webb A, Mackenzie K, Wilson JA, Carding PN (2004). Short, self-report voice symptom scales: psychometric characteristics of the voice handicap index-10 and the vocal performance questionnaire. Otolaryngol Head Neck Surg.

[11] Gillivan-Murphy P, Drinnan MJ, O'Dwyer TP, Ridha H, Carding P (2006). The effectiveness of a voice treatment approach for teachers with self-reported voice problems. J Voice.

[12] Rousseau B, Cohen SM, Zeller AS, Scearce L, Tritter AG, Garrett CG (2011). Compliance and quality of life in patients on prescribed voice rest. Otolaryngol Head Neck Surg.

